# Level of Biogenic Amines in Red and White Wines, Dietary Exposure, and Histamine-Mediated Symptoms upon Wine Ingestion

**DOI:** 10.3390/molecules24193629

**Published:** 2019-10-08

**Authors:** Francesco Esposito, Paolo Montuori, Mario Schettino, Salvatore Velotto, Tommaso Stasi, Raffaele Romano, Teresa Cirillo

**Affiliations:** 1 Department of Agricultural Sciences, University of Naples “Federico II”, via Università, Portici, 100-80055 Naples, Italy; 2 Department of Public Health, University of Naples “Federico II”, via Sergio Pansini, 5-80131 Naples, Italy; 3 Gastroenterology Unit, Department of Clinical Medicine and Surgery, University of Naples “Federico II”, via Sergio Pansini, 5-80131 Naples, Italy; 4 Department of Promotion of Human Sciences and the Quality of Life, University of Study of Roma “San Raffaele”, via di Val Cannuta 247-00166 Roma, Italy; 5 Department of Science and Technology, Newton Consulting srl, 80146 Naples, Italy

**Keywords:** biogenic amines, histamine, wine, dansyl chloride, putrescine, case report

## Abstract

Biogenic amines (BAs) are involved in physiological processes. Foods where typically high levels of BAs occur are fermented food and beverage. This work set out to evaluate the occurrence of BAs in red and white wines, and to also ascertain the dietary exposure to BAs among consumers. Besides, a case report of a probable histamine intoxication upon ingestion of contaminated wine was described. The samples were analyzed through derivatization with dansyl chloride and HPLC-UV detection. Red wines showed higher levels of BAs, especially putrescine (PUT) and histamine (HIS), than white wines (median concentrations of 7.30 and 2.45 mg/L, respectively). However, results of our investigation showed that the dietary exposure to BAs through the consumption of wine (red and white) were lower than the recommended maximum levels for the acute exposure to HIS and tyramine (TYR). In contrast, the levels of BAs in wine on tap were much higher than in bottled wine and close to recommended values. The levels of HIS, TYR, and PUT in tap wine of 9.97, 8.23, and 13.01 mg/L, respectively, were associated with histamine-mediated symptoms in six young individuals after consumption of about three glasses of wine. The overall results and multivariate analysis confirm that red wine shows a higher concentration of BAs than white wine, especially putrescine and histamine. This finding is attributable to the malolactic fermentation that is common for most red wine production. It is also evident that incorrect preservation processes can lead to an increase in BA levels, probably due to the action of bacteria with high decarboxylase activity. The exposure values, although below the toxicity thresholds, could lead to histamine-mediated symptoms in susceptible individuals, also according to the case report discussed in this study.

## 1. Introduction

Biogenic amines (BAs) are low molecular weight non-volatile nitrogenous organic bases, which derive from the decarboxylation of the corresponding amino acids or amination and transamination of aldehydes and ketones [1]. These compounds can be naturally produced either by bacteria during the decarboxylation of amino acids in living cells or formed and degraded as a result of normal metabolic activity in humans, animals, plants, and microorganisms. In strict chemical terms, BAs can be grouped as aliphatic (such as putrescine, cadaverine, spermine, spermidine), aromatic (tyramine, phenylethylamine), and heterocyclic (histamine, tryptamine) [2]. BAs are also grouped according to the number of amino groups and they can be identified as monoamines (phenylethylamine and tyramine) and diamines (cadaverine, putrescine and histamine) [3]. BAs with biological activity are further classified according to their physiological effects on humans, as psychoactive and/or vasoactive. The vasoactive amines are tyramine (TYR), tryptamine, and histamine (HIS), which act on the vascular system; whereas the psychoactive amines such as HIS, putrescine (PUT), and cadaverine (CAD) act on the nervous system instead; PUT and CAD may also enhance the symptoms related to HIS [4,5,6]. BAs can be both essential and harmful to health: They are involved in physiological processes such as blood pressure control, synaptic transmission, allergic response, and cell growth control [7,8]. Some BAs such as PUT, CAD, spermidine, and spermine may react with nitrite and produce carcinogenic compounds (nitrosamines) [9]. HIS and TYR are both the main amines related to adverse health effects; in healthy people, they are degraded through monoaminoxidase (MAO) and diamine oxidase (DAO) enzymes, but the ingestion of a high amount of these BAs may exert specific symptoms. In particular, a “histamine intoxication” may develop as a results of the ingestion of a high amount of food with high levels of HIS [10,11,12,13]. Besides, in some individuals there may be an impairment of DAO’s activity due to genetic predisposition, gastrointestinal diseases, or to the administration of DAO inhibitors leading to an augmented toxicity that leads to symptomatology that resembles an allergic reaction [14]. The latter case is also known as “histamine intolerance” and may even occur after the ingestion of a small amount of HIS. The intoxication is characterized by an incubation period ranging from a few minutes to some hours, with symptoms that last a few hours. Such clinical signs refer to the effects on the blood vessels and smooth muscles and include extrasystoles, migraine, bronchospasm, tachycardia, flushing, and asthma. Dietary HIS and TYR have also been implicated in the pathogenesis of migraines in susceptible individuals suffering from DAO deficiencies [14,15,16]. Foods that may contain high levels of HIS and TYR include fish and products thereof and fermented food products (meat, dairy products, fermented vegetables and sauces, beers and wines) [13,17,18]. Wine, in particular, is often recognized as a cause of foodborne adverse reactions and may exert several symptoms like flushing, itching, headache, meteorism, urticaria, and asthma, which will mimic hypersensitivity to sulfites. As a matter of fact, HIS and other BAs are considered the most important cause for wine intolerance [19,20]. Besides, alcohol is known to reduce DAO activity [13,21,22]. In susceptible individuals, HIS intolerance was triggered by the intake of 4 mg HIS due to the consumption of 0.20 L of sparkling wine containing 20 mg/L of HIS [23]. The most important BAs occurring in foods are PUT, CAD, HIS, TYR, and phenylethylamine, which are the product of the decarboxylation of histidine, tyrosine, ornithine, lysine, and phenylalanine [24,25]. Some BAs, such as PUT and CAD, play an important role in food poisoning as they can enhance the toxicity of HIS [26]. The amount of BAs could also act as a marker of microbiological quality and spoilage level of food [27,28]. Besides, the quantity and type of BA that could develop in the food are highly dependent upon the composition of the product, the bacterial flora, as well as other parameters which influence bacterial growth during food processing and storage and, as a result, BA production can be controlled on various levels during food fermentation [29,30]. Low concentrations of amines occur either in the grape or the must [31,32]; however, the largest contribution in the formation of BAs in the wine should be attributed to yeasts and bacteria. Whilst Buteau et al. (1984) identified the alcoholic fermentation by yeasts as the principal cause of formation of BAs in wine, much of the available literature highlighted the great relevance of lactic acid bacteria (LAB) and secondary fermentation (malolactic fermentation, MLF) in the occurrence of BAs in wine [33,34,35,36]. Finally, a moderate occurrence of BAs in wine is also related to the composition of the soil, use of fertilization, and the poor state-of-health of the grapes. [33,37].

Despite their recognized toxicity and their high content in some fermented products, BAs have not yet been regulated by international law. Up to now, the regulations in force in Europe do not concern wines: the EC regulation 2073/2005 (as well as its amendment EC 1019/2013) sets the food safety criteria for HIS exclusively in fishery products [38,39]. However, some European countries have set maximum permissible values for HIS in wine ranging from 2 to 10 mg/L, but these limits are not mandatory [33]. In 2011, the European Food Safety Authority (EFSA) highlighted that further research was needed on the toxicity and associated concentrations of HIS and TYR, as well as related potentiating effects of PUT and CAD, and the evaluation of the need for the development of new safety criteria for HIS in fermented foods other than fish [13]. Therefore, while many authors dealt with the occurrence of biogenic amines in wines, this study also provides one of the few investigations into the deterministic dietary exposure to HIS and other BAs, through the ingestion of wine, corroborated by the description of a probable HIS intoxication event.

Additionally, the aim of this study was to evaluate the level of PUT, CAD, HIS, and TYR in wine samples and to make a deterministic exposure estimation to these compounds in regular adult consumers. In addition, we have presented a case study involving six young individuals who showed clinical signs of histamine-mediated reaction after the consumption of wine on tap. Furthermore, the incriminated tap wine was sampled and the level of BAs was measured.

## 2. Results and Discussion

**Figure 1:**
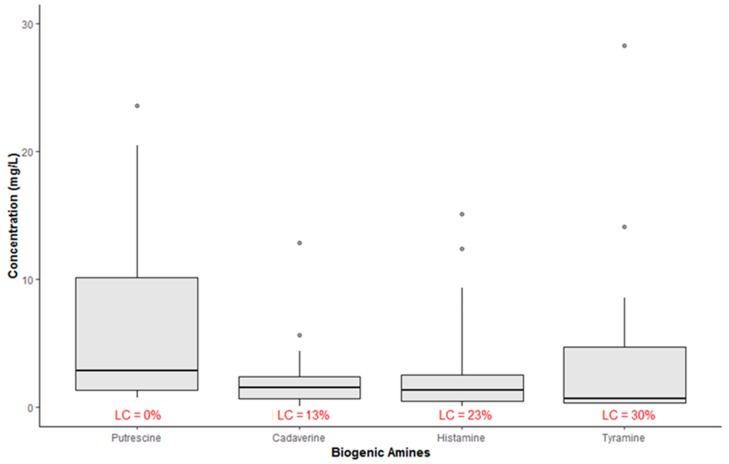
Summary statistic of occurrence of biogenic amines in all wine samples (LC = left-censored values; n = 52).

The boxplots in Figure 1 show the concentration of BAs (mg/L) in all samples. According to this chart, the average concentration values ranged from 2.17 mg/L for CAD to 6.42 mg/L for PUT. Besides, all samples showed quantifiable levels of PUT, while, as regards the other amines, the percentage of left-censored data was in the range 13–30%. 

**Figure 2:**
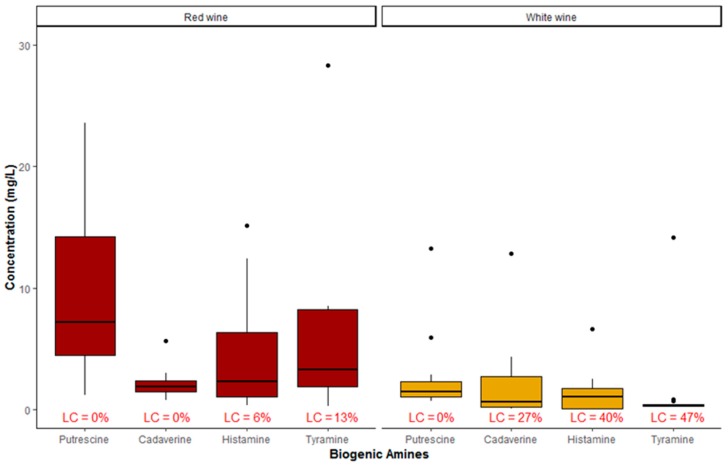
Summary statistic of occurrence of biogenic amines in red and white wines (LC = left-censored values: n = 26).

Considering the two types of red and white separately, Figure 2 shows the summary statistic of BAs detected in the two types of samples. From this chart it is apparent that, as far as white wine is concerned, the highest not aberrant value regarded the CAD with a value of 4.60 mg/L, whereas the lowest median value regarded TYR with a value of 0.31 mg/L. All samples of white wine (100%) showed detectable levels of PUT with a median concentration value of 1.55 mg/L. Red wines showed higher levels of BAs, especially PUT and HIS, than white wines (median concentrations of 7.30 and 2.45 mg/L, respectively). 

Two influential outliers biased the results: a high TYR concentration occurred in a spoiled red wine, whereas higher concentrations of CAD and TYR were detected in sparkling white wine. The underlying causes of these aberrant values could be attributable to spoilage during the storage or to contamination during the secondary fermentation in steel tanks (concerning the white sparkling wine). Hence, these two samples were discarded in the comparison of literature data, but they were considered in the multivariate analysis described in the next sub-section.

A comparison between our data (apart from one strong outlier) and those of other studies is shown in Table 1. Our data are in keeping with those reported by EFSA, 2011, regarding PUT and HIS, and consistent with those reported by Martuscelli et al. (2013) as regards TYR [13,33].

Similarly, Table 2 shows the data related to red wine samples. Between 87% and 100% of the samples were contaminated by one or more BAs. In particular, 100% of the samples showed quantifiable levels of PUT and CAD, and HIS was not detected just in 6% of the samples. Among all BAs, PUT showed the highest concentration, followed by HIS and TYR. 

**Figure 3:**
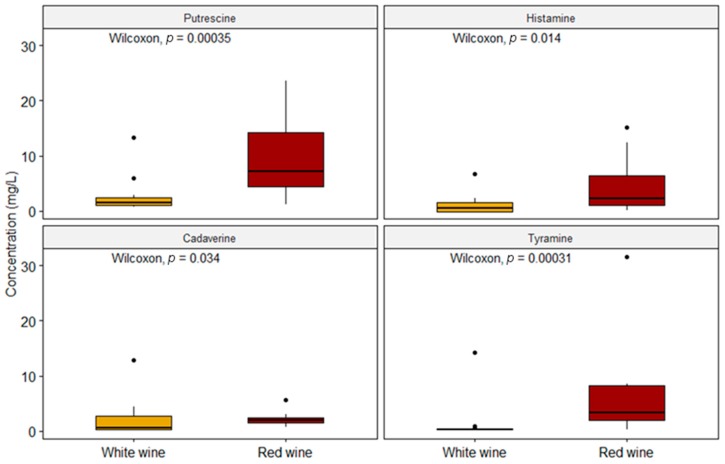
Comparison between the two types of wine with respect to the concentrations of each biogenic amine (n = 52).

Our data are consistent with Martuscelli et al. (2013) as regards PUT, with EFSA (2011) as far as HIS is concerned and with Bover-Cid et al. (2006) as regards TYR (Table 2) [13,33,40]. The increased concentration of PUT in both types of wine is probably due to the predominance of MLF that occurs in red wines by means of some microorganisms such as *Oenococcus oeni*, even though some *Lactobacillus* species can produce PUT during fermentation as well [13,33,41]. In this study, the red wines presented a higher occurrence of BAs than the white wines, both in terms of quantifiable samples and in concentration values. The same remark applies to the results from Bover-Cid et al., 2006, in Spanish wines and from La Torre et al., 2010, in wines from Sicily (Italy), and this also accords with the conclusions by Peña-Gallego et al., 2012, in their review [2,40,42]. As briefly said above, on average, red wines showed, with statistical significance (*p* < 0.05), higher concentrations of the four BAs with the highest contribution of PUT (Figure 3). 

Generally, the “red vinification” is carried out starting from grape skin and pulp, and both of them could release PUT in the must [33]. However, in this case, the higher concentration of PUT could be indeed related to the MLF, as also highlighted in the multivariate analysis described in the following sub-section. 

### 2.1. Principal Components Analysis

A principal component analysis (PCA) was performed to better determine the contribution of variables on the two types of samples. The data were scaled according to the following formula (Pareto scaling):

$$\frac{x_{ij}-\bar{x_{i}}\;}{\sqrt{s_{i}}},$$

$x_{\mathrm{ij}\;}=\;$ Value of the *j*th sample in the *i*th column of the dataset
$\bar{x_{i}}=\;$ Mean value of the *i*th column of the dataset
$s_{i}=$ Standard deviation of the *i*th column of the dataset

**Figure 4:**
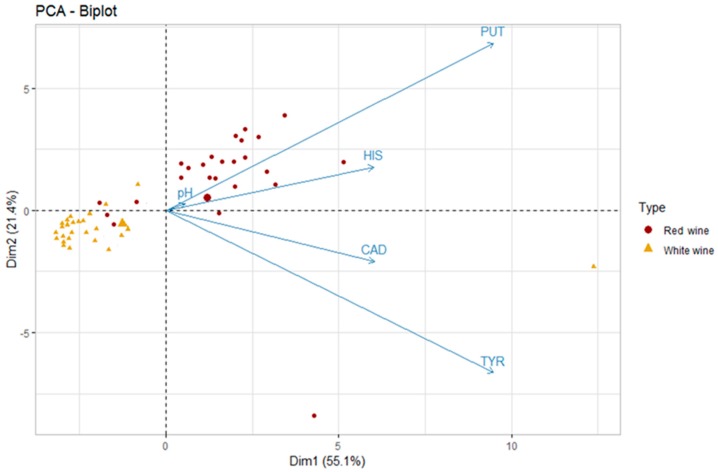
Principal component analysis (PCA) biplot of red and white wines according to the five variables—putrescine (PUT), cadaverine (CAD), histamine (HIS), tyramine (TYR), and pH (the big triangle and circle stand for the mean values of the scores of each type of wine; n = 52).

The first two dimensions of PCA express 76.5% of the total dataset inertia; therefore, the first two components (Dim1 and Dim2) explain the total variability of the dataset. It is apparent from Figure 4 that the two types of wines (red and white) lie in two distinct groups characterized by different values of PUT and HIS and a statistically significant difference in pH values (3.29 for white wines and 3.56 for red wines; Wilcoxon, *p* < 0.05). 

**Figure 5:**
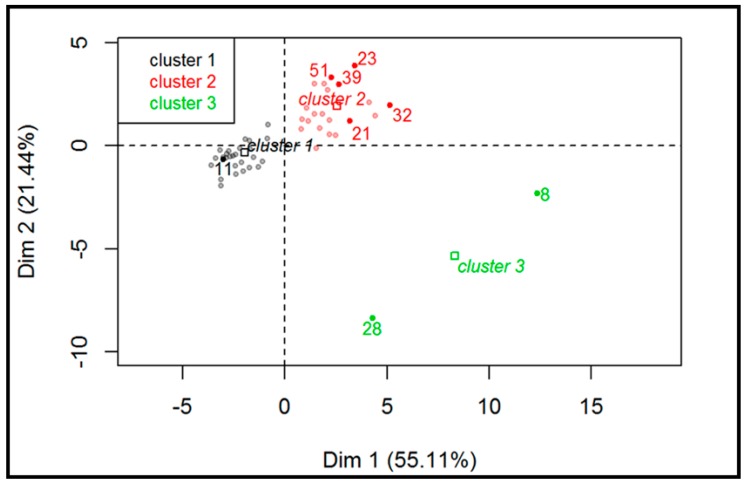
Hierarchical classification of the samples (red and white wines; n = 52).

Figure 5 displays the hierarchical classification of the samples identifying three different clusters. Cluster 1 (grey dots) consists of all white wines and four out of 26 red wines. This group is characterized by low values for the variables PUT, HIS, and TYR, as well as lower pH values. A total of 100% of white wines belong to this cluster and, according to this evaluation, this classification suggests that the difference between white and red wine could be due to the absence of MLF in white (and in some red) wines, as also confirmed by the significant lower pH values. Cluster 2 (red dots) is characterized by red wines that likely underwent MLF or microbial spoilage. Finally, cluster 3 (green dots) is made of two samples characterized by high values for the variables TYR and CAD (variables are sorted from the strongest). These samples were defined as spoiled and included a sparkling sweet white wine (sample #8) and an improperly stored red wine (sample #28).

### 2.2. Exposure to BAs through Consumption of Wine

To evaluate the dietary exposure to BAs through the consumption of wine, the data on wine consumption by Leclerq et al. (2009), across three different age groups, were used [43]. According to these estimates, median adolescent consumers (10–18 years old) drink 0.10 mL/day of wine without gender difference, whereas the consumption rate of male adolescents at the 95th percentile is 40 mL/day and 0.20 mL/day for female peers. As regards adult consumers (18–65 years old), median adult males consume 100.00 mL/day, whereas the 95th consumption is equal to 400.20 mL/day. Adult females consumers drink 20 and 220 mL/day, respectively, as regards median and consumers at the 95th percentile; older males (over 65 years old) consume 166 and 480 mL/day (median and 95th percentile consumers), whereas consumption data of female peers were 60.0 and 280 mL/day. In view of all that has been described so far, two categories of consumers were considered to evaluate the exposure to BAs: consumers at the 50th percentile (median consumers) (Table 3) and consumers at the 95th percentile (high consumers) (Table 4). For each category of exposure, a total of two scenarios were considered: A best case, where the median concentrations of BAs detected in the samples were considered; and a worst case, that took into account the concentrations of BAs at the 95th percentile; the formula used for exposure assessment according to the abovementioned scenarios was:

DI = C×Q

*DI* = Daily intake of BAs (mg/day)
*C* = The 50th and 95th percentile concentration of BAs detected in the samples (mg/L).
*Q* = Individual wine daily consumption of population within different age groups and for median and 95th percentile consumers (L/day).

In the specific case of this study, the body weight was not taken into account as, according to literature data, the recommended maximum levels for the acute exposure to HIS and TYR are expressed in mg per intake, neglecting the anthropometric data.

According to these tables, as regards the BAs that may exhibit acute toxicity levels (HIS and TYR), the exposure scenarios do not entail any critical issue since no value, even among the high consumers, exceeds the levels of acute intoxication (50 mg/meal for HIS and 600 mg/meal for TYR) [13]. However, as reported by Menne et al. (2001), susceptible individuals may develop histamine-mediated symptoms as low as 4 mg per intake [23]. Moreover, the co-presence of PUT and alcohol could lower the toxicity threshold of HIS. Therefore, the exposure values that occur in some groups of high consumers should not be overlooked. 

### 2.3. Case Report

As previously touched upon, the case report described in this study involved six young individuals aged between 22 and 27 years. The subjects, about 3 h after the ingestion of wine, showed the same symptoms, namely headache, flushing, dizziness, nausea, and altered systolic blood pressure (144 ± 8 mm Hg) and an average pulse rate of 92 ± 6 bpm; some individuals showed gastrointestinal disorders as well (i.e., abdominal cramps and diarrhea). The subjects were admitted to the emergency department at a local hospital and routine laboratory tests, as well as cardiac enzymes, were all in the reference ranges. During the anamnesis, the patients reported that they attended a dinner with 25 friends and the onset of symptoms occurred within 3 h after the ingestion of a moderate amount of wine (about three glasses) and none of them ingested fish or cheese during the last 24 h. The students were discharged with a diagnosis of drunkenness after receiving treatment with IV fluids and metoclopramide with partial relief of symptoms. 

Nevertheless, after 24 h, the patients had not recovered their normal state of health and some symptoms persisted, whereas a full clinical recovery was achieved after 60 h for all of the patients. The wine, responsible for this symptomatology among the students, had previously been sampled for this study, and it was immediately analyzed. The samples were divided into two aliquots and each of them was analyzed in triplicate. The pH of the wine was 3.46 ± 0.03 and the ethanol concentration was 10.5% *v*/*v*. The microbiological analysis was negative for the most common pathogens, whereas the chemical analysis revealed not negligible levels of the following BAs: PUT, CAD, HIS, and TYR. The concentration of these compounds is shown in Table 5. 

From the analysis of a seemingly clear case of drunkenness, the persistence of symptoms over 48 h induced one of the patients to evaluate the safety of the ingested product and that in the first place caused symptoms indeed compatible with slight alcohol intoxication. The levels of HIS that have been detected, even below the toxicity threshold of 50 mg, did not rule out the possibility that the cause of the symptoms could depend primarily upon a HIS-related syndrome that was initially overlooked. The co-presence of alcohol as well as other diamines like PUT and CAD could lower the threshold of HIS toxicity interacting with the MAO and DAO action, leading to HIS intolerance. It is, hence, essential to consider that symptoms related to the ingestion of HIS, while being more common as a result of consumption of tuna or mackerel, may well be related to the ingestion of other fermented foods, especially if alcohol is also present, and although the level of HIS does not exceed the toxic threshold [13]. Literature data show that, in susceptible individuals, the intake of 4 mg of HIS through a glass of wine may exert obvious clinical symptoms of HIS poisoning in the 30% of patients involved in a double-blind design [23]. In this case, the individuals ingested about 5 mg of HIS through the consumption of sparkling wine with high levels of PUT as well. 

## 3. Materials and Methods 

### 3.1. Sampling and Extraction

A total of 52 samples of red and white wine equally distributed were selected and were purchased at local markets. The procedures for the extraction of the different food matrices were carried out according to Preti et al. (2015) [44]. Briefly, 25 mL of the sample were acidified with HClO_4_ 10.3 M to a final concentration of 0.2 M and the internal standard (IS) (1,7-diaminoeptane) was added to reach a final concentration of 0.8 mg/L. Subsequently the sample was derivatized according to Chiacchierini et al. (2006) with some minor modifications [45]. 

### 3.2. Chemicals and Reagents

Perchloric acid (70%), acetone (analytical grade), water, and acetonitrile (HPLC grade), as well as the other reagents, were supplied by Sigma-Aldrich (Milan, Italy). The BA standards (PUT, CAD, HIS, and TYR), the derivatizing reagent dansyl chloride, and the internal standard 1,7-diaminoeptane (IS) were all supplied by Supelco, (Bellefonte, PA, USA). A stock solution of 2 g/L was prepared by diluting the four BAs with HPLC grade acidified water (HCl 0.1 M). The standard solutions were stored in the dark at 4 °C and freshly prepared every 30 days.

The calibration curve was built on the basis of six standard solutions containing a complete mix of the four BAs. The standard solutions were obtained by diluting aliquots of the stock solution to a final volume of 25 mL before adding HClO_4_ 10.3 M in an amount such to get a final acid concentration of 0.2 M in the sample. After the derivatization procedure described in the following paragraph, the standard mix was injected in a concentration range between 0.5 and 8.0 mg/L of each BA. The calibration curve was plotted as the peak area ratio of each BA to the IS derivative versus concentration.

### 3.3. Derivatization

A total of 1.0 mL of the sample taken from the previously acidified 25 mL was added to 200 µL of NaOH 2 M (until pH 11), 300 µL of saturated NaHCO_3_ solution, and 2 mL of dansyl chloride solution in acetone (10 mg/mL daily prepared). After stirring, the samples were left in the dark at 45 °C for 60 min, and the excess of dansyl chloride was neutralized by adding 100 µL of NH_4_OH 25% (*v*/*v*). The final volume was brought to 5 mL by adding acetonitrile. The dansylated amine solution obtained was filtered through a 0.22 µm Sartorius filter and analyzed with HPLC-UV in triplicate.

### 3.4. Chromatographic Conditions

The samples were analyzed with an HPLC system with binary pump ATVP LC-10, equipped with UV-Vis detector (Shimadzu, Kyoto, Japan). The analytical column was a Supelco C18 (250 mm, ID 4.6 mm I.D., particle size 2.6 µm). The temperature of the analytical column was maintained at 25 °C and the mobile phase was made of water and acetonitrile (ACN) according to the following gradient: From 40% of ACN to 80% in 25 min, and 100% during the next 5 min and finally back to 40% during the last 5 min, maintaining this percentage until the end of the run whose total time was 50 min. The UV wavelength was set at 254 nm. The sampling refresh was 10 Hz. 

### 3.5. Recoveries, Determination and Quantification Limits

The recoveries were determined by spiking six wine samples (red and white) in triplicate at a concentration of 0.5, 1.0, and 2.0 mg/L of each BA. The recovery values were 96 ± 4, 92 ± 4, 92 ± 3, and 89 ± 4, respectively, for PUT, CAD, HIS, and TYR. 

The limit of detection (LOD) and the limit of quantification (LOQ) were calculated, respectively, using the standard deviation of the response (σ) and the slope of the calibration curve (S) according to the following formulas:

$$\mathrm{LOD}=3.3\;\frac{\sigma }{S}\;,$$

LOQ=3 LOD .

LOQ values were equal to 0.12, 0.12, 0.15, and 0.30 mg/L, respectively, for PUT, CAD, HIS, and TYR. Left-censored data were considered according to an upper bound approach as equal to LOQ.

Finally, the intra-day and inter-day repeatability were assessed through injection of the standards at two different concentration levels (0.5 and 1.0 mg/L) five times during a day (intra-day) and seven consecutive days (inter-day). The intra-day repeatability was expressed as the relative standard deviation (RSD) and ranged from 2.9% to 4.2%, whereas the inter-day repeatability was always below 5%.

### 3.6. Statistical Analysis

Data analysis and graph processing were performed using R Software version 3.6.0 [46], using the following packages: FactoMinerR, factoextra, FactoInvestigate, ggplot2, and ggpubr [47,48,49,50,51].

### 3.7. Case Study

This paper also describes a particular case study which involved six young healthy students aged between 22 and 27 years. These subjects showed symptoms compatible with HIS intoxication after drinking sparkling red wine on tap during dinner and an aliquot of this product had been previously sampled for this study. The sample was bought at a wine shop in the province of Naples, and each subject consumed about three glasses (450 mL). The onset of symptoms was within 8 h after the consumption and some of these symptoms persisted for the next 24 h.

## 4. Conclusions

The results obtained confirm that red wine (n = 26) presents higher concentrations of BAs than white wine (n = 26). This finding is attributable to the MLF that is common for most red wine production. It is also evident that incorrect preservation processes can lead to an increase in the levels of BAs, probably brought about by the action of bacteria with high decarboxylase activity.

The exposure values found in this study, although lower than the toxicity thresholds widely documented in the literature, in susceptible individuals could lead to symptoms compatible with HIS intolerance. The toxicological data that are currently available for HIS are the result of experiments with healthy volunteers and sensitive people. These results are not always reproducible due to intra- and inter-individual variations in susceptibility. Changes in sensitivity may also be the result of interaction with other BAs, other components such as alcohol, or drugs like MAO and DAO inhibitors. Such uncertainties can lead to an underestimation of the adverse effect in susceptible persons and future works should focus on the determination of the real risk of acute toxicity for consumers. Finally, clinicians should take into account the likelihood of a differential diagnosis of HIS poisoning among patients who show compatible symptoms after the ingestion of fermented food or alcoholic beverages.

In view of what has been illustrated in this work and due to the lack of legal limits in wines, it would be desirable to conduct further studies on the effects of HIS in synergy with other BAs and in the co-presence of alcohol, in order to assess the need of establishing any legal limit in wines, to safeguard the health of the consumers and the quality of the finished product at once.

## Figures and Tables

**Table 1:** Comparison of biogenic amines levels with literature data on white wines (mg/L).

BA	This Study			EFSA, 2011 [13]		Tuberoso et al. (2014) [32]	Martuscelli et al. (2013) [33]	Bover-Cid et al. (2006) [40]
	Mean	Median	95th pctl	Mean	95th pctl	Mean	Mean	Mean
**Putrescine**	1.85	1.55	3.96	1.4–1.5	3.9–4.4	5.96	2.24	4.00
**Cadaverine**	1.49	0.92	4.36	0.1–0.2	0.3–0.4	2.06	0.79	0.10
**Histamine**	0.76	0.28	2.41	0.8–0.9	2.6	ND	0.18	0.20
**Tyramine**	0.38	0.31	0.78	1.1–1.2	4.3–4.5	NQ	0.41	0.20

BA = Biogenic amine; EFSA = European Food Safety Authority; Pctl = percentile; ND = Not detectable; NQ = Not quantifiable.

**Table 2:** Comparison of biogenic amines levels with literature data on red wines (mg/L).

BA	This Study			EFSA, 2011 [13]		Tuberoso et al. (2014) [32]	Martuscelli et al. (2013) [33]	Konakovsy et al. (2011) [19]	Bover-Cid et al. (2006) [40]
	Mean	Median	95th pctl	Mean	95th pctl	Mean	Mean	Median	Mean
**Putrescine**	9.98	7.30	20.31	4.2–4.8	9.5–11.5	20.50	7.88	19.4	27.90
**Cadaverine**	1.71	1.93	4.25	0.2–0.5	0.6–1.6	2.13	0.11	0.58	0.20
**Histamine**	2.36	2.45	9.32	3.6–3.7	12.3–12.4	6.61	2.91	7.20	3.90
**Tyramine**	3.43	3.20	8.24	2.7–2.9	7.8–8.5	9.06	5.22	3.52	3.30

BA = Biogenic amine; EFSA = European Food Safety Authority; Pctl = percentile; ND = Not detectable; NQ = Not quantifiable.

**Table 3:** Exposure to biogenic amines in consumers at 50th percentile (median consumers) (mg/day).

Gender	BA	Adolescent (10–18 Aged)		Adult (18–65 Aged)		Elderly (>65 Aged)	
		Best Case	Worst Case	Best Case	Worst Case	Best Case	Worst Case
Males	PUT	≤0.001	0.002	0.279	1.808	0.463	3.002
	CAD	≤0.001	≤0.001	0.155	0.437	0.257	0.725
	HIS	≤0.001	≤0.001	0.128	0.932	0.212	1.547
	TYR	≤0.001	≤0.001	0.062	0.842	0.103	1.397
Females	PUT	≤0.001	0.002	0.056	0.363	0.167	1.085
	CAD	≤0.001	≤0.001	0.031	0.088	0.093	0.262
	HIS	≤0.001	≤0.001	0.026	0.187	0.077	0.559
	TYR	≤0.001	≤0.001	0.012	0.169	0.037	0.505

PUT = putrescine; CAD = cadaverine; HIS = histamine; TYR = tyramine.

**Table 4:** Exposure to biogenic amines in consumers at the 95th percentile (high consumers) (mg/day).

Gender	BA	Adolescent (10–18 Aged)		Adult (18–65 Aged)		Elderly (>65 Aged)	
		Best Case	Worst Case	Best Case	Worst Case	Best Case	Worst Case
Males	PUT	0.112	0.723	1.116	7.237	1.339	8.680
	CAD	0.062	0.175	0.619	1.747	0.743	2.096
	HIS	0.051	0.373	0.512	3.729	0.614	4.473
	TYR	0.025	0.337	0.248	3.368	0.297	4.040
Females	PUT	≤0.001	0.004	0.613	3.978	0.781	5.063
	CAD	≤0.001	≤0.001	0.340	0.960	0.433	1.222
	HIS	≤0.001	0.002	0.282	2.050	0.358	2.609
	TYR	≤0.001	0.002	0.136	1.852	0.173	2.357

PUT = putrescine; CAD = cadaverine; HIS = histamine; TYR = tyramine.

**Table 5:** Levels of biogenic amines detected in the wine responsible for intoxication.

Compound	Concentration (mg/L)
Putrescine	13.01 ± 0.30
Cadaverine	1.51 ± 0.17
Histamine	9.97 ± 0.21
Tyramine	8.23 ± 0.22
